# Diets Partially Replaced With Cassava Residue Modulate Antioxidant Capacity, Lipid Metabolism, and Gut Barrier Function of Huanjiang Mini-Pigs

**DOI:** 10.3389/fvets.2022.902328

**Published:** 2022-05-17

**Authors:** Md. Abul Kalam Azad, Huijiao Jiang, Hengjia Ni, Yating Liu, Pan Huang, Jun Fang, Xiangfeng Kong

**Affiliations:** ^1^Hunan Provincial Key Laboratory of Animal Nutritional Physiology and Metabolic Process, CAS Key Laboratory of Agro-Ecological Process in Subtropical Region, National Engineering Laboratory for Pollution Control and Waste Utilization in Livestock and Poultry Production, Institute of Subtropical Agriculture, Chinese Academy of Sciences, Changsha, China; ^2^University of Chinese Academy of Sciences, Beijing, China; ^3^Hunan Engineering Laboratory for Pollution Control and Waste Utilization in Swine Production, College of Bioscience and Biotechnology, Hunan Agricultural University, Changsha, China

**Keywords:** alternative feed, cytokines, hepatic biochemistry, intestinal microbiota, gut physiology, pigs

## Abstract

Agricultural by-products have been identified as potential feed resources in animal production. The present study investigated the effects of cassava residue (CR) or fermented CR (FCR) on antioxidant capacity, immunity, gut barrier functions, and lipid metabolism in pigs. A total of 120 healthy Huanjiang mini-piglets were assigned into three groups, including control group (basal diet), CR group (basal diet + 5% CR), and FCR group (basal diet + 5% FCR). The experiment lasted for 30 days. The results showed that, dietary CR or FCR supplementation increased the jejunal catalase (CAT, *P* = 0.063) and glutathione peroxidase (GSH-Px, *P* < 0.05) levels and hepatic superoxide dismutase (SOD, *P* < 0.05) level while decreased (*P* = 0.077) ileal malondialdehyde (MDA) level, when compared with the control group. Dietary CR supplementation increased intestinal SOD and hepatic GSH-Px levels, whereas decreased jejunal and hepatic MDA levels (*P* < 0.05). Dietary CR supplementation increased the levels of secretory immunoglobulin A (sIgA) in the intestine and liver, as well as jejunal interleukin (IL)-10, ileal tumor necrosis factor (TNF)-α, and hepatic interferon (IFN)-γ, whereas dietary CR or FCR supplementation decreased the jejunal IL-1β level and increased hepatic IL-10 level (*P* < 0.05). In the intestinal microbiota analysis, dietary CR or FCR supplementation enhanced the colonic α-diversity and ileal *Actinobacteria* abundance, whereas decreased ileal *Verrucomicrobia* and colonic *Tenericutes* abundances (*P* < 0.05). In addition, dietary FCR supplementation increased *Firmicutes* and decreased *Bacteroidetes* abundances in the ileum and colon, whereas CR supplementation increased *Escherichia-Shigella* and decreased *Terisporobacter* abundances in the ileum (*P* < 0.05). Moreover, dietary CR or FCR supplementation up-regulated (*P* < 0.05) the gene expressions related to gut barrier functions of piglets. However, dietary CR supplementation showed negative impacts on hepatic lipid metabolism by up-regulating the expression of genes associated with fatty acid synthesis and triglyceride and lipid metabolism. In conclusion, dietary CR or FCR supplementation can maintain the health of piglets by increasing antioxidant capacity, gut barrier function, and altering the intestinal microbiota composition, but CR supplementation may increase the potential risk of abnormal lipid metabolism.

## Introduction

The constant growth of the population is closely related to a growing demand for food of plant and animal origin. In addition, with the improvement of people's living standards and health awareness, consumers' demand for high-quality and safe pork is increasing. Feeding strategies in livestock production, particularly non-conventional native feed resources and cost-effective alternatives, have been gained particular interest to improve the sustainable development of animal industries. Recent research evidence has confirmed that cereal-based conventional concentrates could be partially replaced with non-conventional feed resources as livestock feed with no detrimental on animal health and production performance (1, 2). Moreover, non-conventional feed resources present beneficial functions, including antimicrobial activity, antioxidant activity, and gut health (3, 4). Cassava (*Manihot esculenta*) is widely grown in tropical and subtropical regions. The world-wide production of cassava is estimated to be 276.7 million tons, and it provides valuable food sources for more than 105 developing countries (5, 6). Cassava is still widely used in the production of starch, bioethanol, and other bio-products, such as feed, medicines, and biopolymers (5). However, after processing cassava products, a large number of residues remained unused and may cause environmental pollution. Cassava residue (CR) contains a higher content of calories and various contents of proteins, ether extract, mineral substances, and vitamins that can make it highly nutritious compared with other tubers. Therefore, the application of CR to pigs' feed could help reduce its environmental impact and the waste of nutrients.

Extensive research evidence highlighted that microbial fermentation could improve the nutritional quality of pig feed by increasing the bioavailability of nutrients and reducing the content of anti-nutritional factors (7–9). Recent studies have found that fermented corn-soybean meal could improve the immune function of pigs, and fermented feed has been shown positive impacts on the gut functions of pigs by enhancing the beneficial bacteria (*Lactobacillus*) and inhibiting the pathogenic bacteria (*Escherichia coli*) in the intestine (10). Furthermore, fermented feed supplements from apple pomace, mushroom, mulberry leaf extracts, etc., have been extensively studied to improve reproductive performance, growth performance, antioxidant capacity, and intestinal function of pigs (11–14). However, the application of CR or fermented CR (FCR) in pig feed is still limited.

Thus, it remains uncertain whether CR or FCR boosts intestinal functions and gut microbiota composition, as well as the lipid metabolism of piglets. Moreover, our previous study also showed that microbial fermentation could enhance the nutritional quality of CR by increasing crude protein and crude fat contents and decreasing the detergent fiber content (15). Therefore, we hypothesized that partial replacement of corn-soybean bean with CR or FCR might influence the antioxidant capacity, intestinal gut ecosystem, and hepatic lipid metabolism of piglets. In the present study, we evaluated the effects of CR or FCR supplements on the intestinal and hepatic antioxidant capacity, gut barrier function, intestinal microbiota composition, and lipid metabolism of Huanjiang mini-piglets.

## Materials and Methods

### Preparation of Cassava Residue and Fermented Cassava Residue

The integral CR was provided by Du'an Honghe Starch Co., Ltd. (Guangxi, China) and contained 16.13 MJ/kg gross energy, 2.73% crude protein, 37.78% neutral detergent fiber, and 2.52% minerals. A 200 kg of CR was mixed with 20 kg corn-soybean-based meal thoroughly, and then 0.20 kg of fermented liquid (*Lactobacillus plantarum* 1 × 10^8^ + *Bacillus subtilis* 0.2 × 10^8^ + *Saccharomyces cerevisiae* 0.2 × 10^8^) was sprayed on the surface of feed and mixed vigorously. After mixing, CR was packed and sealed in a polyethylene bag with a capacity of 5 kg at 27–30°C for 7 days. Finally, FCR samples were stored at 4°C to prevent deterioration. The selection and concentration of the bacterial species were based on our previous findings (15). Fermentation liquid broth was obtained from Hunan Lifeng Biotechnology Co., Ltd. (Changsha, China).

### Experimental Design and Diet Management

A total of 120 healthy Huanjiang mini-piglets with similar body weight (BW, 8.85 ± 0.64 kg) were selected and assigned randomly to one of three groups with eight replicates per group and five piglets per replicate. The three groups were as follows: (a) control group, fed a basal diet; (b) CR group, fed a basal diet supplemented with 5% CR; and (c) FCR group, fed a basal diet supplemented with 5% FCR. Dietary CR and FCR were mixed uniformly with the basal diet and fed twice daily (08:00 and 15:00). During the experimental period, piglets were housed in pens (2 × 3 m), and the rearing house had forced ventilation and maintained the temperature at 23–26°C. The supplementing dose of the CR and FCR were based on our previous findings (unpublished data). The composition and nutrients levels of the basal diet for piglets met the Chinese local swine nutrient requirements (NY/T65-2004) and the National Research Council (NRC, 2012) diet requirements (16, 17). The composition and nutrient levels of the basal diet for piglets are presented in Supplementary Table 1. The experiment lasted for 30 days. Feed and drink were freely accessible during the experimental period.

### Sample Collection

At the end of the 30-day trial, one piglet from each replicate and a total of eight piglets per group (*n* = 8) with similar BW was selected for sample collection. The piglets were then euthanized using electric shock (120 V, 200 Hz) as previously described (18). The ileum (10 cm above the ileocecal junction) and colon (middle portion) segments were collected and immediately kept at −20°C for microbiota community analyses. The jejunum and ileum tissues were excised, flushed in ice-cold phosphate buffer solution, and then scrapped by glass slides. The mucosa scrapings (~2 g) were sampled, quickly frozen into liquid nitrogen, and immediately stored at −80°C for further studies of antioxidant, secretory immunoglobulin A (sIgA), cytokines, and gut barrier function. The liver tissue samples were collected, snap frozen into liquid nitrogen, and then stored at −80°C for further analyses of biochemical parameters, antioxidant, sIgA, cytokines, and lipid metabolism.

### Hepatic Biochemical Parameter Assays

Hepatic biochemical parameters, including triglycerides (TG), total cholesterol (TC), alanine aminotransferase (ALT), alkaline phosphate (ALP), high-density lipoprotein-cholesterol (HDL-C), and low-density lipoprotein-cholesterol (LDL-C) were determined using commercially available kits (F. Hoffmann-La Roche Ltd., Basel, Switzerland) and Roche automatic biochemical analyzer (Cobas c311, F. Hoffmann-La Roche Ltd., Basel, Switzerland).

### Antioxidant Capacity Assays

Approximately 100 mg of frozen intestinal (jejunum and ileum) and hepatic tissue samples were thawed and quickly homogenized with ice-cold physiologic saline (1:9, w/v). Then the samples were centrifuged at 2,000 × *g* for 20 min at 4°C. The supernatants were used for further analysis. The oxidant and antioxidant indicators, including malondialdehyde (MDA), catalase (CAT), superoxide dismutase (SOD), glutathione (GSH), and GSH peroxidase (GSH-Px), were analyzed by commercially available ELISA assay kits (Mei mian, Jiangsu, China) according to the manufacturer's instructions with a Multiscan Spectrum Spectrophotometer (Tecan, Infinite M200 Pro, Switzerland).

### Intestinal and Hepatic Secretory IgA and Cytokines Assays

Samples (100 mg) from the jejunum, ileum, and hepatic tissues were homogenized with ice-cold physiological saline (1:9, w/v) and centrifuged at 2,000 × *g* for 20 min at 4°C. Then the supernatants were collected to measure the levels of sIgA and cytokines, including interleukin (IL)-1β, IL-10, IL-22, tumor necrosis factor (TNF)-α, and interferon (IFN)-γ by commercially available ELISA assay kits (Mei mian, Jiangsu, China) according to the manufacturer's instructions.

### Intestinal Microbiota Analysis

The intestinal bacterial genomic DNA was extracted from ileal and colonic samples (*n* = 7–8) with a Hipure Stool DNA Kit (Megan, Guangzhou, China) according to the manufacturer's protocol. The extracted DNA concentration and purity were determined using NanoDrop ND-1000 spectrophotometer (NanoDrop Technologies Inc., Wilmington, USA) and 0.80% agarose gel electrophoresis, respectively. The bacterial 16S rRNA genes from the V3-V4 region were amplified by polymerase chain reaction (PCR) with the forward primer 338F (5′-ACTCCTACGGGAGGCAGCA-3′) and reverse primers 806R (5′-GGACTACHVGGGTWTCTAAT-3′). The PCR components and PCR amplification conditions were maintained in accordance with the manufacturer's guidelines (New England Biolab Inc., MA, USA). The amplified PCR products were detected with 2.0% agarose gel electrophoresis with an AXYGEN AxyPrepDNA gel recovery kit. According to the standard protocol, purified amplicons were pooled into equimolar and pair-end (2 × 300) sequencing by the MiSeq Reagent Kit in an Illumina MiSeq platform (Illumina, San Diego, USA) to construct a sequence library by the Biomarker Technologies Co., Ltd. (Beijing, China). Raw sequence data of the present study are deposited in the NCBI Sequence Read Archive (SRA) with under the accession number PRJNA793624.

The α-diversity of the OTU level was measured by the quantitative insights into microbial ecology (QIIME, version 1.8) software. The β-diversity analysis was used to evaluate the structural difference of ileal and colonic microbiota in the control and treatment groups. Partial least squares discriminant analysis (PLS-DA) and nonmetric multidimensional scaling (NMDS) were also performed to find the structural variation of microbial communities among the three groups. Using Metastats (http://metastats.cbcb.umd.edu/) analysis procedure, the abundances of different taxa at the phylum and genus levels were determined among the three groups. Finally, the linear discriminant analysis to estimate the effect size (LEfSe) analysis was performed to evaluate the different abundant taxa using default parameters.

### Hepatic Lipid Metabolism and Gut Barrier Function-Related Gene Expression Analysis

According to the manufacturers' instructions, total RNA from the intestinal and hepatic tissues was isolated using TRIzol (Invitrogen, Carlsbad, USA). Approximately 1000 ng of total RNA was reversely treated into cDNA by a PrimeScrip RT Reagent Kit with gDNA Eraser (TaKaRa Biotechnology, Dalian, China). After that, 2 μL of cDNA template was added to a total volume of 10 μL RT-PCR reaction system solution containing 0.25 μL of each forward and reverse primers, following the 5.0 μL SYBR Green mix, and 2.5 μL of deionized distilled water. The PCR cycle conditions were as follows: an initial step at 95°C for 5 min, followed by 40 cycles of denaturation at 95°C for 5 s, and annealing at 60°C for 30 s. Pig-specific primer sequences for lipid metabolism and gut barrier function related genes are presented in Supplementary Table 2. The mRNA expression level for each gene was calculated using the 2^−Δ*ΔCt*^ method.

### Statistical Analysis

The intestinal and hepatic parameters, including biochemical parameters, antioxidant capacity, sIgA, and cytokines, as well as the gene expression levels related to lipid metabolism and gut barrier functions were analyzed by one-way analysis of variance. The comparative analysis among different groups was performed using Tukey's *post-hoc* test (SPSS 26.0; SPSS Inc., Chicago, IL, USA). The correlation between the gut barrier functions-related indexes and intestinal microbial genera was measured using Spearman's correlation by the R package. All data are presented as means ± standard error of the mean (SEM), and data were considered statistically significant when *P* < 0.05, and trends when 0.05 ≤ *P* < 0.10.

## Results

### Effects of CR or FCR on Hepatic Biochemical Parameters of Piglets

The effects of dietary CR and FCR supplementation on hepatic biochemical parameters are shown in Table 1. Dietary FCR supplementation increased (*P* < 0.05) the hepatic levels of TG and ALP and tended to decrease (*P* = 0.073) the hepatic LDL-C level of piglets compared with the control and CR groups. However, dietary CR or FCR supplementation had no impact (*P* > 0.05) on the hepatic TC, ALT, and HDL-C levels.

**Table 1:** Effects of cassava residue (CR) or fermented cassava residue (FCR) on hepatic biochemical parameters of piglets.

**Items**	**Control group**	**CR group**	**FCR group**	**SEM**	***P*-values**
ALT (U/L)	446.40	418.28	408.44	17.38	0.660
ALP (U/L)	196.60^b^	186.67^b^	308.00^a^	17.82	0.004
HDL-C (mmol/L)	0.07	0.08	0.06	0.01	0.792
LDL-C (mmol/L)	0.01	0.01	<0.01	0.00	0.073
TC (mmol/L)	0.69	0.80	0.88	0.04	0.220
TG (mmol/L)	0.16^b^	0.21^b^	0.31^a^	0.02	<0.001

*Data are expressed as means with their SEM (n = 8). Means within a row without a common superscript letter are different (P < 0.05)*.

*ALP, alkaline phosphate; ALT, alanine aminotransferase; HDL-C, high-density lipoprotein-cholesterol; LDL-C, low-density lipoprotein-cholesterol; TC, total cholesterol; TG, triglycerides*.

### Effects of CR or FCR on Intestinal and Hepatic Antioxidant Capacity of Piglets

The effects of dietary CR or FCR supplementation on antioxidant activity of piglets are shown in Table 2. In the jejunum, dietary CR supplementation increased the SOD and decreased MDA activities of piglets (*P* < 0.05), whereas dietary CR or FCR increased (*P* < 0.05) the GSH-Px activity and displayed a trend for an increased (*P* = 0.063) CAT activity of piglets, when compared with the control group. In the ileum, the activities of CAT, GSH, and SOD were increased (*P* < 0.05) in the CR group compared with the control and FCR groups. Moreover, the GSH-Px activity was tended to increase (*P* = 0.056) in the CR group compared with the other two groups, whereas MDA level was tended to decrease (*P* = 0.077) in the CR and FCR groups compared with the control group. In the liver, the GSH-Px activity was higher and MDA level was lower in the CR group compared with the control group, whereas SOD activity was higher in the CR and FCR groups compared with the control group (*P* < 0.05).

**Table 2:** Effects of cassava residue (CR) or fermented cassava residue (FCR) on intestinal and hepatic antioxidant capacity of piglets.

**Items**	**Control group**	**CR group**	**FCR group**	**SEM**	***P*-values**
**Jejunum**					
CAT (U/mg)	3.81	4.72	4.26	0.15	0.063
GSH (μmol/mg)	4.78	5.51	5.28	0.22	0.433
GSH-Px (U/mg)	24.43^b^	38.29^a^	36.28^a^	1.74	<0.001
MDA (nmol/mg)	3.62^a^	2.46^b^	2.96^ab^	0.18	0.026
SOD (U/mg)	94.62^b^	164.86^a^	119.53^ab^	9.75	0.006
**Ileum**					
CAT (U/mg)	5.00^b^	6.82^a^	5.53^b^	0.19	<0.001
GSH (μmol/mg)	5.43^b^	7.71^a^	5.51^b^	0.30	<0.001
GSH-Px (U/mg)	45.09	60.62	46.45	3.01	0.056
MDA (nmol/mg)	4.43	3.69	3.58	0.79	0.077
SOD (U/mg)	124.10^b^	251.38^a^	183.75^b^	15.07	0.001
**Liver**					
CAT (U/mg)	3.26	4.14	3.63	0.17	0.120
GSH (μmol/mg)	4.02	4.84	4.46	0.19	0.209
GSH-Px (U/mg)	16.24^b^	26.30^a^	24.53^ab^	1.81	0.040
MDA (nmol/mg)	2.93^a^	2.10^b^	2.44^ab^	0.14	0.042
SOD (U/mg)	269.02^b^	447.29^a^	431.33^a^	24.47	0.001

*Data are expressed as means with their SEM (n = 8). Means within a row without a common superscript letter are different (P < 0.05)*.

*CAT, catalase; GSH, glutathione; GSH-Px, GSH peroxidase; MDA, malondialdehyde; SOD, superoxide dismutase*.

### Effects of CR or FCR on Intestinal and Hepatic Secretory IgA and Cytokines Levels of Piglets

The effects of dietary CR or FCR supplementation on the intestinal and hepatic sIgA and cytokines levels of piglets are presented in Table 3. In the jejunal mucosa, the sIgA concentration was increased (*P* < 0.05) in the CR group compared with the control and FCR groups. In addition, the IL-1β concentration in the CR and FCR groups was tended to decrease (*P* = 0.067), whereas IL-10 concentration in the CR group was tended to increase (*P* = 0.069), when compared with the control group. In the ileal mucosa, sIgA and IL-10 concentrations were increased in the CR group compared with the control and FCR groups, whereas IL-1β concentration was decreased in the FCR group compared with the control and CR groups (*P* < 0.05). Moreover, the TNF-α concentration had a trend to increase (*P* = 0.053) in the CR group compared with the control and FCR groups. In the liver, sIgA and IFN-γ concentrations in the CR group and the IL-10 concentration in the CR and FCR groups were increased (*P* < 0.05) compared with the control group.

**Table 3:** Effects of cassava residue (CR) or fermented cassava residue (FCR) on intestinal and hepatic secretory IgA and cytokine levels of piglets.

**Items**	**Control group**	**CR group**	**FCR group**	**SEM**	***P-*values**
**Jejunum**					
IL-1β (ρg/mg)	217.46	181.50	188.09	6.84	0.067
IL-10 (ρg/mg)	47.64	58.11	52.89	1.88	0.069
IL-22 (ρg/mg)	84.57	82.52	92.25	4.60	0.680
TNF-α (ρg/mg)	50.75	50.35	50.71	2.42	0.998
IFN-γ (ρg/mg)	11.32	11.69	10.51	0.50	0.628
sIgA (μg/mg)	13.43^b^	18.06 ^a^	14.03^b^	0.62	0.001
**Ileum**					
IL-1β (ρg/mg)	280.19^a^	276.63^a^	236.24^b^	7.52	0.020
IL-10 (ρg/mg)	63.18^b^	84.61^a^	63.36^b^	3.25	0.003
IL-22 (ρg/mg)	119.12	120.02	108.89	4.78	0.586
TNF-α (ρg/mg)	69.51	79.38	65.57	2.50	0.053
IFN-γ (ρg/mg)	14.60	15.34	13.90	0.54	0.555
sIgA (μg/mg)	16.76^b^	22.40^a^	16.19^b^	0.81	<0.001
**Liver**					
IL-1β (ρg/mg)	170.71	154.01	179.90	7.17	0.342
IL-10 (ρg/mg)	34.45^b^	49.48^a^	47.16^a^	2.10	0.003
IL-22 (ρg/mg)	58.57	61.43	66.32	2.96	0.579
TNF-α (ρg/mg)	40.59	46.22	40.95	2.46	0.599
IFN-γ (ρg/mg)	8.24^b^	12.10^a^	10.97^ab^	0.61	0.022
sIgA (μg/mg)	11.11^b^	15.05^a^	12.90^ab^	0.57	0.011

*Data are expressed as means with their SEM (n = 8). Means within a row without a common superscript letter are different (P < 0.05)*.

*IFN, interferon; IL, interleukin; TNF, tumor necrosis factor; sIg, secretory immunoglobulin A*.

### Effects of CR or FCR on the Intestinal Microbial Diversity of Piglets

**Figure 1:**
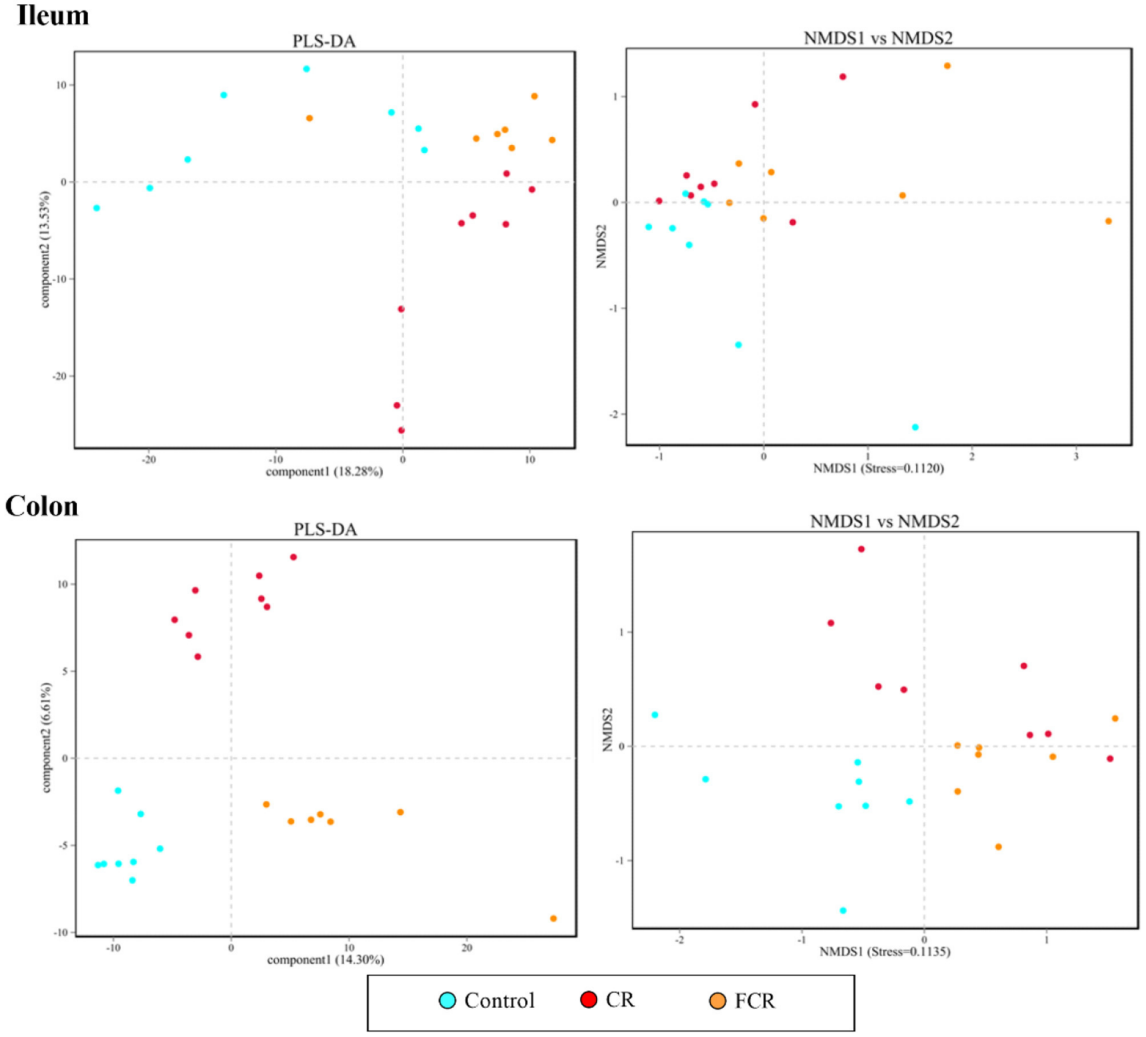
Partial least squares discriminant analysis (PLS-DA) and nonmetric multidimensional scaling (NMDS) analyses of the bacterial community in the ileum and colon of piglets. Each symbol represents the gut microbiota from one piglet, 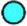 represents the piglets in the control group, 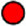 represents the piglets in the CR group, and 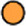 represents the piglets in the FCR group (*n* = 7–8).

The Miseq sequencing data obtained from the ileum and colon were merged, quality controlled, and then clarified with an illusion illustration. The analyses of OTUs and species taxonomy were performed on the optimized sequence. The OTUs' α-diversity indexes are presented in Table 4. There were no significant differences in the ileal α-diversity indexes except an increasing trend of the raw tags (*P* = 0.099) in the CR and FCR groups compared with the control group. In the colon, the OTU, ACE, and Chao1 indexes were increased (*P* < 0.05) in the CR and FCR groups compared with the control group. Moreover, the FCR group had a higher (*P* < 0.05) coverage index in the colon compared with the CR group. The results of PLS-DA and NMDS (Figure 1) showed that the intestinal bacterial β-diversity of the control, CR, and FCR groups were tended to cluster into three groups. In addition, there was more clear separation of the PLS-DA and NMDS in the colon than in the ileum.

**Table 4:** Effects of cassava residue (CR) or fermented cassava residue (FCR) on microbial diversity indices in the ileum and colon of piglets.

**Item**	**Control group**	**CR group**	**FCR group**	**SEM**	***P*-values**
**Ileum**					
Raw tags	78,693.13	79,063.50	78,917.29	72.91	0.099
Effective tags	75,764.00	76,724.75	74,037.14	635.47	0.237
OTU	560.00	567.00	480.00	21.12	0.194
ACE	622.13	612.26	547.97	17.97	0.208
Chao1	618.55	622.04	557.63	16.95	0.243
Simpson	0.10	0.16	0.14	0.02	0.598
Shannon	3.61	3.33	3.01	0.17	0.407
Coverage	0.998925	0.999	0.998686	0.00	0.533
**Colon**					
Raw tags	79,052.00	78,966.88	78,957.50	57.71	0.778
Effective tags	70,473.50	70,015.25	69,953.75	418.67	0.869
OTU	444.00^b^	501.75^a^	535.75^a^	11.52	0.001
ACE	507.77^b^	576.84^a^	582.08^a^	10.03	0.001
Chao1	534.13^b^	582.81^a^	588.66^a^	9.33	0.024
Simpson	0.11	0.08	0.06	0.01	0.308
Shannon	3.70	3.72	3.98	0.11	0.488
Coverage	0.998375^ab^	0.998188^b^	0.998537^a^	0.00	0.04

*Data are expressed as means with their SEM (n = 7–8). Means within a row without a common superscript letter are different (P < 0.05)*.

### Effects of CR or FCR on the Intestinal Microbial Community of Piglets

**Figure 2:**
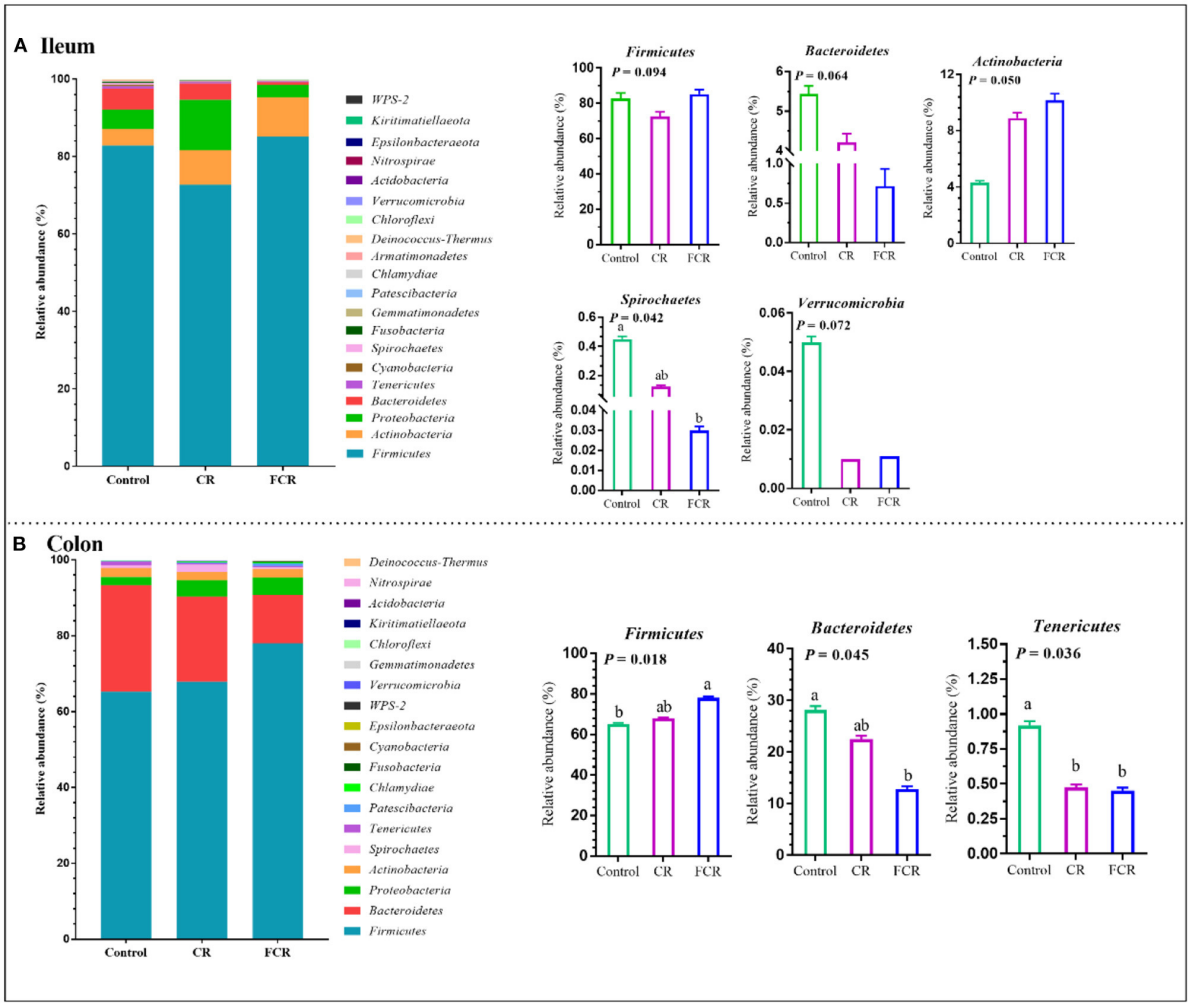
The relative abundance of piglets' ileal **(A)** and colonic **(B)** microbiota community and taxonomic differences at the phylum level (*n* = 7–8). The data are presented as the means ± SEM, and values with different letters mean significant difference (*P* < 0.05).

The effects of dietary CR or FCR supplementation on the intestinal microbiota community composition were assessed using taxon-dependent analysis. The microbiota community composition in the ileum and colon at the phylum level are shown in Figure 2. In the ileum, *Firmicutes* (72.71–85.16%), *Actinobacteria* (4.31–10.17%), *Proteobacteria* (3.22–13.08%), and *Bacteroidetes* (0.60–5.44%) were the top four abundant phyla of the different treatment groups (Figure 2A). The relative abundances of *Bacteroidetes* (*P* = 0.064) and *Spirochaetes* (*P* < 0.05) were lower in the FCR group compared with the control group. The relative abundance of *Actinobacteria* showed an upward trend (*P* = 0.050), while *Verrucomicrobia* presented a downward trend (*P* = 0.072) in the CR and FCR groups compared with the control group. Moreover, the relative abundance of *Firmicutes* had a downward trend (*P* = 0.094) in the CR group compared with the other two groups (Figure 2A). In the colon, *Firmicutes, Bacteroidetes, Proteobacteria*, and *Actinobacteria* were the top four abundant phyla and accounted for 65.23% to 78.04%, 12.77% to 28.13%, 2.13% to 4.57%, and 2.24% to 2.41%, respectively (Figure 2B). The relative abundance of *Firmicutes* was increased (*P* < 0.05) and *Bacteroidetes* was decreased (*P* < 0.05) in the FCR group compared with the control group. In addition, the relative abundance of *Tenericutes* was decreased (*P* < 0.05) in the CR and FCR groups compared with the control group (Figure 2B).

**Figure 3:**
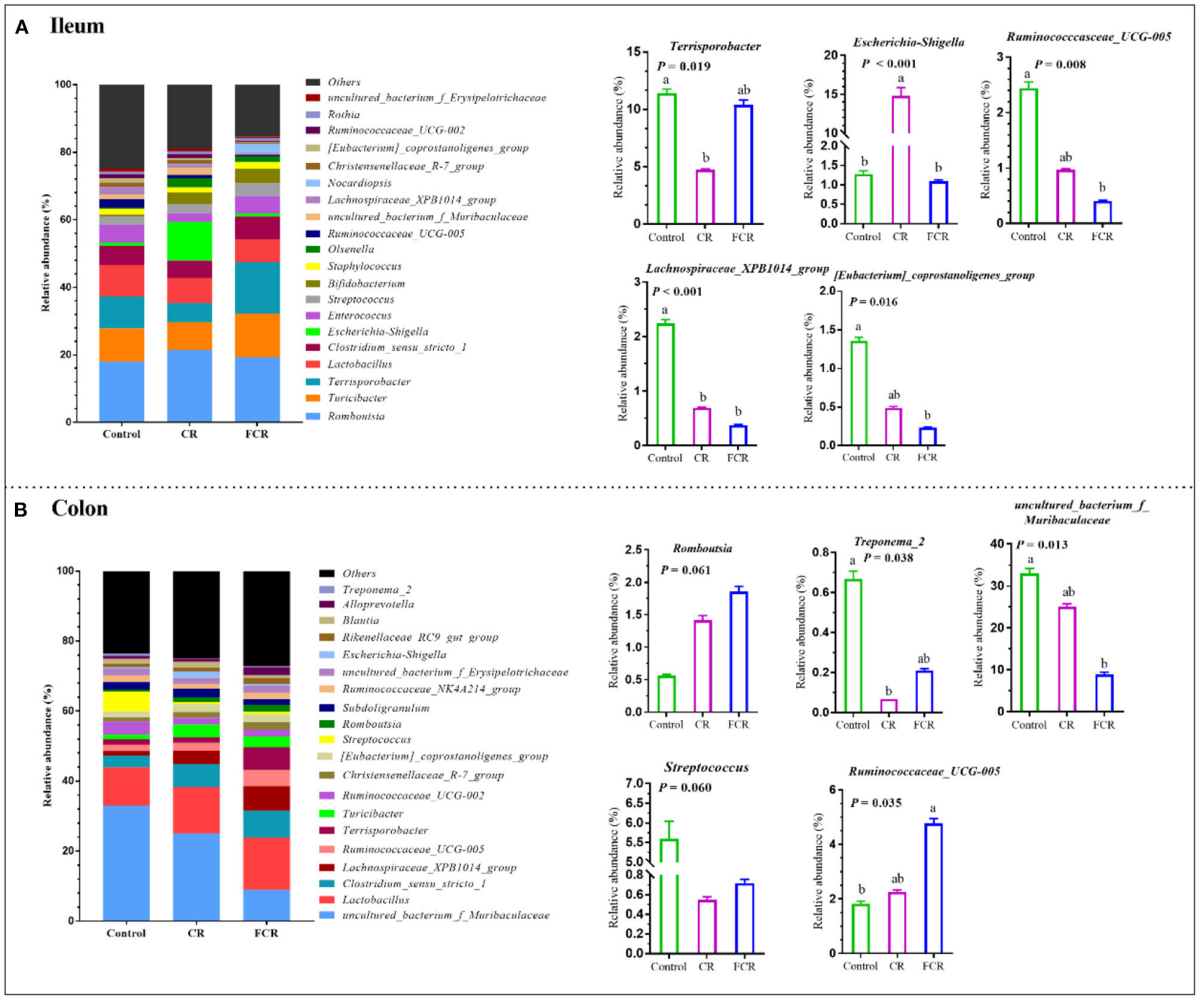
The relative abundance of piglets' ileal **(A)** and colonic **(B)** microbiota community and taxonomic differences at the genus level (*n* = 7–8). The data are presented as the means ± SEM, and values with different letters mean significant difference (*P* < 0.05).

The 20 most abundant bacteria in the ileum and colon at the genus level are presented in Figure 3. In the ileum, the most five dominant genera were *Romboutsia* (18.03–21.43%), *Turicibacter* (8.25–12.81%), *Terrisporobacter* (5.71–15.26%), *Lactobacillus* (6.86–9.33%), and *Clostridium_sensu_stricto_1* (0.98–11.48%) (Figure 3A). Compared with the control group, the relative abundances of *Ruminnococcaceae_UCG-005* and *[Eubacterium]_coprostanoligens_group* were decreased (*P* < 0.05) in the FCR group. The relative abundance of *Terrisporobacter* in the CR group and *Lachnospiraceae_XPB1014_group* in the CR and FCR groups were decreased (*P* < 0.05) compared with the control group. In addition, the relative abundance of *Escherichia-Shigella* was higher in the CR group compared with the other two groups (Figure 3A). In the colon, *uncultured_bacterium_f_Muribaculaceae* (8.97–33.01%), *Lactobacillus* (10.92–14.98%), *Clostridium_sensu_stricto_1* (3.46–7.94%), *Lanchospiraceae_XPB_group* (1.20–6.99%), and *Ruminococcaceae_UCG-005* (1.82–4.77%) were the most five dominant genera (Figure 3B). The relative abundance of *uncultured_bacterium_f_Muribaculaceae* was decreased while the *Ruminococcaceae_UCG-005* was increased in the FCR group compared with the control group (*P* < 0.05). In addition, the relative abundance of *Treonema_2* was decreased (*P* < 0.05) in the CR group compared with the control group. Furthermore, the relative abundance of *Romboutsia* had an upward trend (*P* = 0.061), while the *Streptococcus* presented a downward trend (*P* = 0.060) in the CR and FCR groups compared with the control group (Figure 3B).

**Figure 4:**
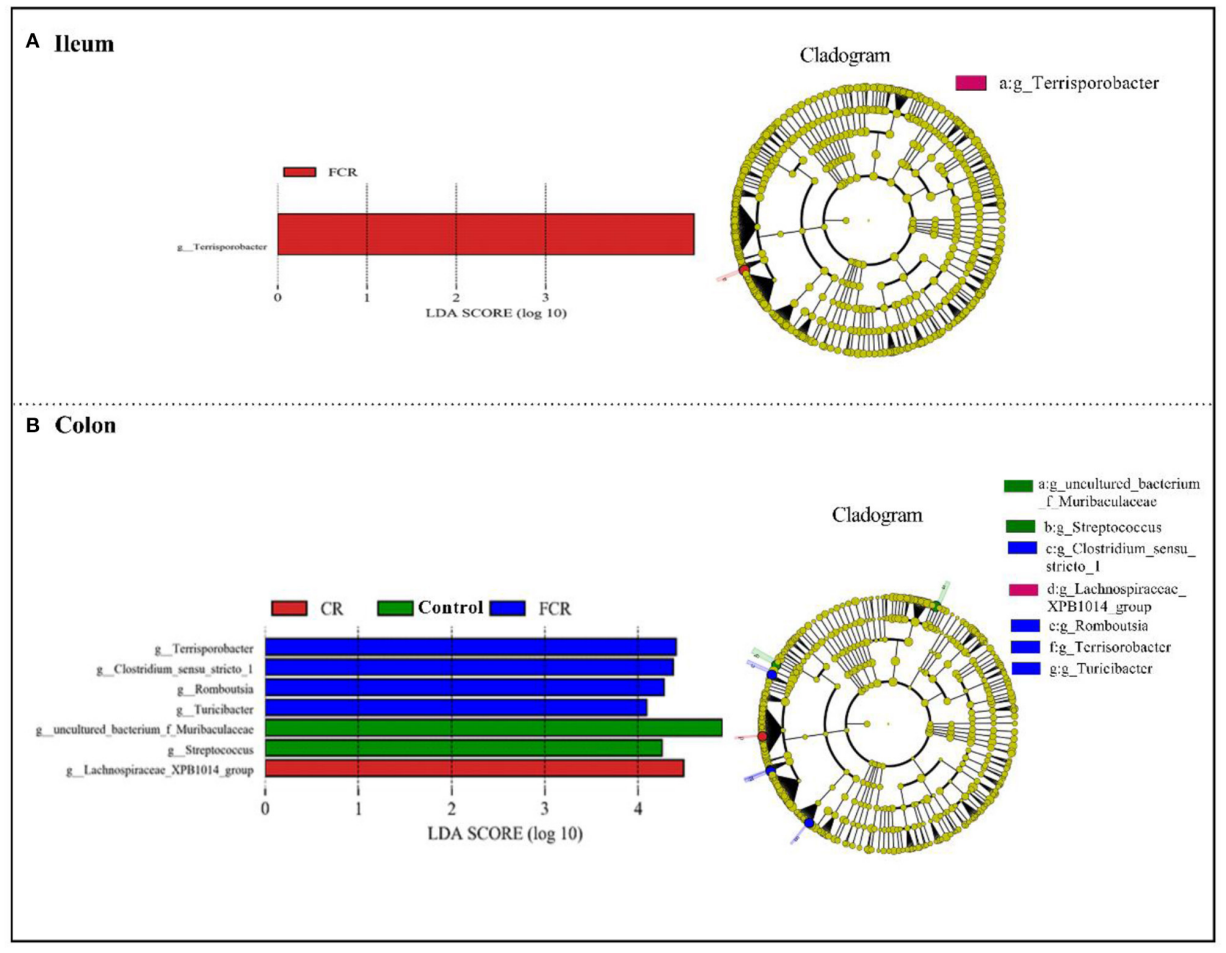
Linear discriminant analysis combined effect size (LEfSe) measurement analysis of microbiota in the ileal **(A)** and colonic **(B)** contents of piglets.

To further investigate the differences of ileal and colonic microbiota communities among the three groups, LEfSe analysis (LDA threshold score ≥ 4.0) was carried out at the genus level (Figure 4). There was a significant enrichment of *Terrisporobacter* in the ileum of the FCR group (Figure 4A). In the colon, *uncultured_bacterium_f_Muribaculaceae* and *Streptococcus* were enriched in the control group, whereas *Lachnospiraceae_XPB1014_group* was enriched in the CR group. Moreover, *Terrisporobacter, Clostridium_sensu_stricto_1, Romboutsia*, and *Turicibacter* were enriched in the FCR group (Figure 4B).

### Effects of CR or FCR on Gene Expressions Related to Gut Barrier Function of Piglets

The effects of dietary CR or FCR supplementation on gene expressions related to gut barrier function of piglets are presented in Table 5. Compared with the control group, dietary CR or FCR supplementation up-regulated (*P* < 0.05) the mRNA expressions of *claudin-1, occludin*, and *mucin-1* in the jejunum of piglets. The jejunal mRNA expressions of *E-cadherin, Nrf2*, and *CD36* were up-regulated while the *TLR4* was down-regulated in the CR group, compared with the control and FCR groups (*P* < 0.05). Moreover, the mRNA expression of *NF-*κ*B* was up-regulated (*P* < 0.05) and *Keap1* was down-regulated (*P* < 0.05), and *ZO-1* had a toward of up-regulating trend (*P* = 0.087) in the jejunum of the FCR group compared with the control and CR groups. In the ileum, the mRNA expressions of *claudin-1, E-cadherin, NF-*κ*B*, and *Nrf2* were up-regulated in the CR and FCR groups compared with the control group, whereas *NF-*κ*B* was up-regulated and *E-cadherin* was down-regulated in the FCR group compared with the CR group (*P* < 0.05). Furthermore, compared with the control group, the mRNA expressions of *mucin-1, TLR4*, and *CD36* were up-regulated in the CR group, while *occludin* and *Keap1*were up-regulated in the FCR group (*P* < 0.05).

**Table 5:** Effects of cassava residue (CR) or fermented cassava residue (FCR) on gut barrier function-related gene expressions of piglets.

**Item**	**Control group**	**CR group**	**FCR group**	**SEM**	***P*-values**
**Jejunum**					
*ZO-1*	0.99	1.02	1.25	0.05	0.087
*Claudin-1*	0.94^b^	1.15^a^	1.23^a^	0.04	0.002
*Occludin*	0.98^b^	1.16^a^	1.22^a^	0.03	<0.001
*Mucin-1*	0.98^b^	1.19^a^	1.26^a^	0.03	<0.001
*E-cadherin*	1.00^b^	2.05^a^	1.01^b^	0.12	<0.001
*Keap1*	1.43^a^	1.35^a^	1.32^b^	0.02	0.047
*NF-κB*	1.43^b^	1.40^b^	1.66^a^	0.04	0.006
*TLR4*	1.35^a^	1.14^b^	1.42^a^	0.03	<0.001
*Nrf2*	1.11^b^	2.14^a^	1.07^b^	0.12	<0.001
*CD36*	1.01^b^	2.05^a^	1.03^b^	0.11	<0.001
**Ileum**					
*ZO-1*	1.00	1.01	0.99	0.01	0.875
*Claudin-1*	0.99^b^	1.21^a^	1.36^a^	0.04	<0.001
*Occludin*	1.00^b^	1.01^b^	1.28^a^	0.03	<0.001
*Mucin-1*	1.00^b^	1.24^a^	1.02^b^	0.03	0.002
*E-cadherin*	1.00^c^	1.14^a^	1.05^b^	0.02	<0.001
*Keap1*	1.00^b^	1.07^b^	1.28^a^	0.03	<0.001
*NF-κB*	1.00^c^	1.15^b^	1.27^a^	0.03	<0.001
*TLR4*	1.00^b^	1.21^a^	1.05^b^	0.02	<0.001
*Nrf2*	1.00^b^	1.24^a^	1.26^a^	0.03	<0.001
*CD36*	1.00^b^	1.25^a^	1.05^b^	0.03	<0.001

*Data are expressed as means with their SEM (n = 8). Means within a row without a common superscript letter are different (P < 0.05)*.

*Keap1, kelch-like ECH-associated protein 1; NF-κB, nuclear factor kappa B; Nrf2, nuclear factor erythroid 2-related factor 2; TLR4, toll like receptor 4; ZO-1, zonula occludens-1*.

### Correlation Analysis of Microbial Abundances and Gut Barrier Function

**Figure 5:**
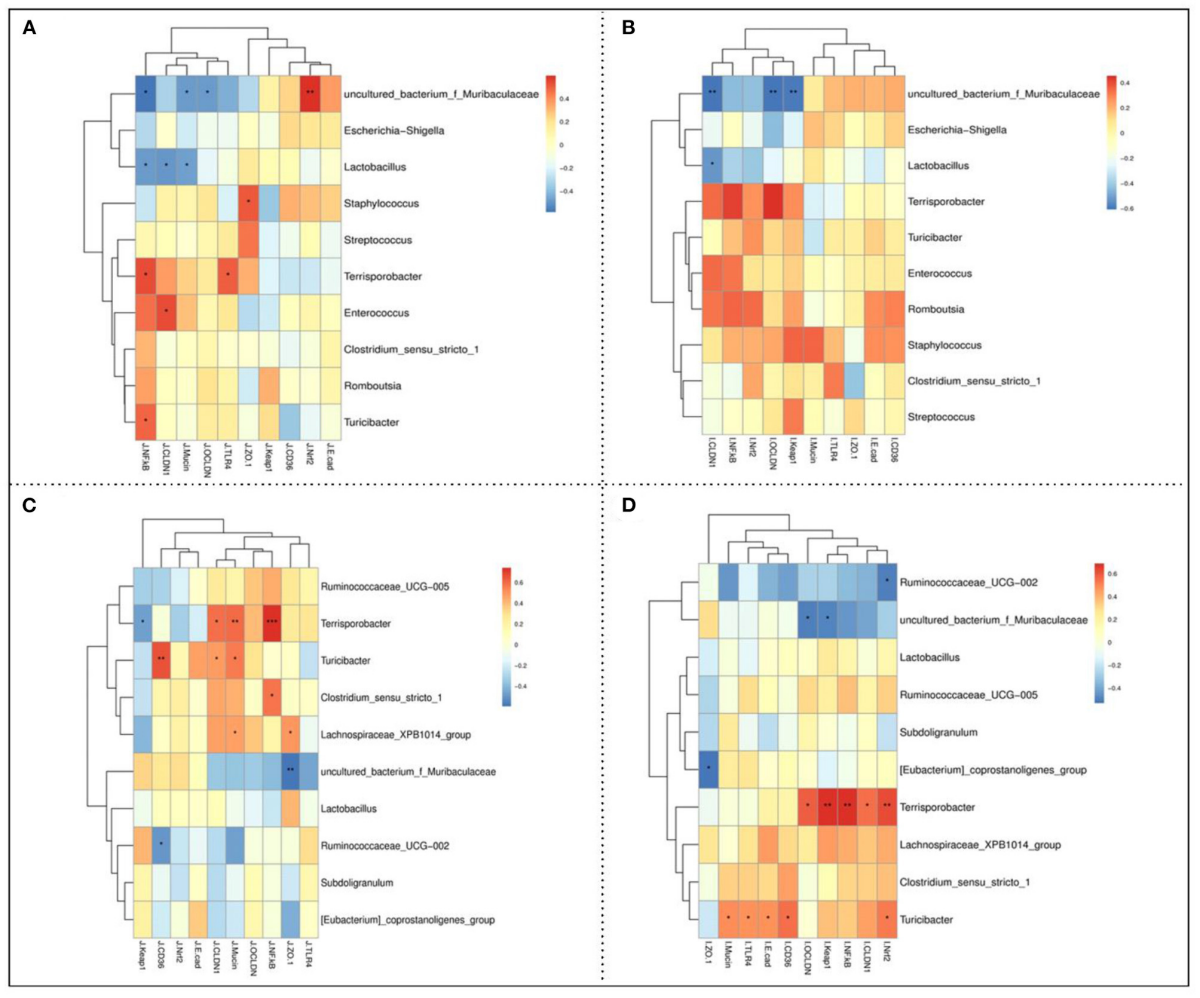
Spearman's correlation between the jejunal **(A)** and ileal **(B)** barrier function-related parameters and ileal microbiota abundances, and jejunal **(C)** and ileal **(D)** barrier function-related parameters and colonic microbiota abundances. * *P* < 0.05, ** *P* < 0.01, and *** *P* < 0.001.

The Spearman's correlation analysis was assessed to evaluate the correlations between the expression levels of gut barrier function-related genes and the intestinal microbiota relative abundance at the genus level of piglets. As shown in Figure 5A, the ileal *uncultured_bacterium_f_Muribaculaceae* abundance was positively correlated with the jejunal *Nrf2* while negatively correlated with the jejunal *occludin, claudin 1*, and *NF-*κ*B* mRNA expression levels. The ileal *Lactobacillus* abundance was negatively correlated with the jejunal *mucin, claudin 1*, and *NF-*κ*B* mRNA expression levels. Moreover, there were positive correlations between ileal *Staphylococcus* abundance with the jejunal *ZO-1, Terrisporobacter* abundance with the jejunal *TLR4* and *NF-*κ*B, Enterococcus* abundance with the *claudin1*, and *Tucibacter* abundance with the jejunal *NF-*κ*B* mRNA expression levels. Furthermore, the ileal *uncultured_bacterium_f_Muribaculaceae* abundance was negatively correlated with the ileal *keap1, occludin*, and *claudin 1* mRNA expression levels, while the ileal *Lactobacillus* abundance was negatively correlated with the ileal *occludin 1* mRNA expression level (Figure 5B). As shown in Figure 5C, colonic *Terrisporobacter* abundance was positively correlated with the jejunal *NF-*κ*B, mucin*, and *claudin 1*, while negatively correlated with the *keap1* mRNA expression levels. In addition, there were also positive correlations between the colonic *Turicibacter* abundance with the jejunal *mucin, claudin1*, and *CD36, Clostridium_sensu_stricto_1* abundance with the jejunal *NF-*κ*B*, and *Lachnospiraceae_XPB1014_group* abundance with the jejunal *ZO-1* and *mucin* mRNA expression levels. Moreover, the colonic *uncultured_bacterium_f_Muriculceae* and *Ruminococcaceae_UCG-002* abundances were negatively correlated with the jejunal *ZO-1* and *CD36* mRNA expression levels, respectively. As shown in Figure 5D, colonic *Turicibacter* abundance was positively correlated with the ileal *mucin, TLR4, E-cadherin, CD36*, and *Nrf2* mRNA expression levels, while colonic *Ruminococcaceae_UCG-002* abundance was negatively correlated with the ileal *Nrf2* mRNA expression level. Moreover, colonic *Terrisporobacter* abundance was positively correlated with the ileal *occludin, keap1, NF-*κ*B, claudin1*, and *Nrf2*, while colonic *uncultured_bacterium_f_Muribaculaceae* abundance was negatively correlated with the ileal *occludin* and *keap1* mRNA expression levels.

### Effects of CR or FCR on Gene Expressions Related to Hepatic Lipid Metabolisms of Piglets

**Figure 6:**
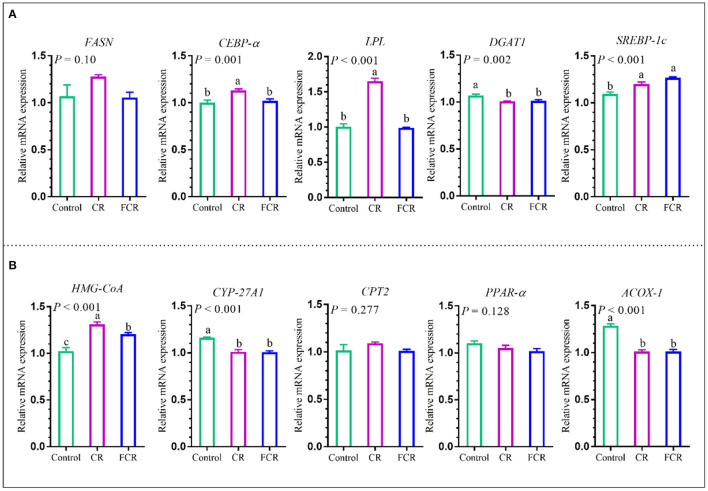
Effects of cassava residue (CR) or fermented cassava residue (FCR) on hepatic lipid metabolism of piglets. The mRNA expression levels of enzymes associated with fatty acid synthesis and triglyceride metabolism **(A)** and cholesterol metabolism **(B)**. The data are presented as the means ± SEM, and values with different letters mean significant difference (*P* < 0.05). *ACOX-1*, acetyl-CoA oxidase 1; *CEBP-*α, CCAAT enhancer-binding protein alpha; *CPT2*, carnitine palmitoyl transferase 2; *CYP-27A1*, cytochrome p540 family 27 sub-family A member 1; *DGAT1*, diacylglycerol-o-acyltransferase 1; *FASN*, fatty acid synthase; *HMG-CoA*, 3-hydroxy-3-methylglutaryl-CoA reductase; *LPL*, lipoprotein lipase; *PPAR-*α, peroxisome proliferator activated receptor alpha; *SREBP-1c*, sterol regulatory element-binding protein 1c.

The effects of dietary CR or FCR supplementation on the mRNA expression levels of hepatic enzymes associated with fatty acid synthesis and triglyceride metabolism, such as fatty acid synthase (*FASN*), CCAAT enhancer-binding protein alpha (*CEBP-*α), lipoprotein lipase (*LPL*), diacylglycerol-o-acyltransferase 1 (*DGAT1*), and sterol regulatory element-binding protein 1c (*SREBP-1c*) are presented in Figure 6A. The mRNA expression levels of *CEBP-*α and *LPL* were up-regulated (*P* < 0.05) in the CR group compared with the control and FCR groups. Compared with the control group, the mRNA expression level of *DGAT1* was down-regulated (*P* < 0.05) and *SREBP-1c* was up-regulated (*P* < 0.05) in the CR and FCR groups. However, there was no significant difference (*P* > 0.05) in the mRNA expression level of *FASN* among the three groups.

The effects of dietary CR or FCR supplementation on the mRNA expression levels of hepatic enzymes associated with cholesterol metabolism are presented in Figure 6B. The hepatic cytochrome p450 family 27 sub-family A member 1 (*CYP-27A1*) and acetyl-CoA oxidase 1 (*ACOX-1*) mRNA expression levels were down-regulated (*P* < 0.05), whereas 3-hydroxy-3-methylglutaryl-CoA reductase (*HMG-CoA*) was up-regulated (*P* < 0.05) in the CR and FCR groups compared with the control group. In addition, hepatic *HMG-CoA* mRNA expression level was down-regulated (*P* < 0.05) in the FCR group compared with the CR group. However, no significant differences (*P* > 0.05) were observed in the hepatic carnitine palmitoyl transferase 2 (*CPT2*) and peroxisome proliferator activated receptor alpha (*PPAR-*α) mRNA expression levels among the three groups.

## Discussion

Agricultural industries produce a large quantity of agricultural by-products after processing and production, which are rich sources of bioactive compounds, such as polysaccharides, flavonoids, proteins, vitamins, and mineral substances. These compounds have been found to improve the antioxidant capacity and gut barrier functions and gut microbiota alteration. Therefore, the present study evaluated the partial replacement of corn-soybean meal with CR or FCR on intestinal and hepatic antioxidant capacity, immunomodulatory function, gut microbiota composition, gut barrier functions, and lipid metabolism of piglets. In the present study, dietary CR or FCR supplementation improved the intestinal and hepatic antioxidant capacity and immunomodulatory function, gut barrier function, and regulated hepatic lipid metabolism of Huanjiang mini-pigs.

The MDA is known as a decomposition product of lipoperoxidation and the important marker of oxidative stress (19). In addition, it has been found that early weaning stress could induce oxidative stress by increasing the levels of MDA and free radicals (e.g., H_2_O_2_ and OH^∙^) and impairing the cellular antioxidant defense system in piglets (20). Thus, it is possible to reduce the degree of lipid destruction and increase the ability of ROS scavenging by lowering the MDA level. In the present study, dietary CR supplementation decreased the MDA level in the jejunum and liver, and had a decreasing trend in the ileum, suggesting that dietary CR supplementation may influence oxidant status of piglets. Moreover, the SOD, GSH-Px, and CAT are the major GSH-dependent enzymatic antioxidants and have strong free radicals scavenging ability (21). The GSH is a major endogenous antioxidant, acting as a free radical scavenger in the cell, whereas GSH-Px is the speed-limiting enzyme and an important indicator of oxidative stress. The present study showed that dietary CR or FCR supplementation increased the GSH-Px activity in the jejunum and SOD activity in the liver of piglets, while dietary CR increased the SOD activity in the jejunum and ileum and GSH-Px activity in the liver of piglets. In addition, dietary CR supplementation increased the CAT and GSH activities in the ileum of piglets. Therefore, these findings revealed that dietary CR or FCR supplementation could elevate the antioxidant capacity of piglets which may contribute to reduce oxidative stress in the early age of pigs.

The inflammatory cytokines have been found to disrupt gut barrier function. For example, excessive pro-inflammatory cytokines (i.e., IL-1β, TNF-α, and IFN-γ), level cause immune response disorders, which further can lead to inflammation, while anti-inflammatory cytokines (such as IL-10) can reduce inflammation (22). In the present study, dietary CR supplementation increased the concentrations of IL-10 in the jejunum, IL-10 and TNF-α in the ileum, and IFN-γ in the liver while both CR and FCR supplementation decreased IL-1β in the jejunum and increased IL-10 in the liver. The decreased pro-inflammatory cytokines and increased anti-inflammatory cytokines in piglets fed with CR or FCR may be partly improved the piglet's immunomodulatory functions. In addition, the present study showed that dietary CR supplementation increased the intestinal and hepatic sIgA secretion in the piglets. Secretory IgA is the most important antibody in mucosal secretions and has a strong ability to prevent the pathogen invasion from protecting the mucosal barrier system (23). The possible reason for the beneficial impacts of dietary CR or FCR on immune responses is that the fiber contents presented in the CR could directly increase the potential immune cell activation and cell-mediated immunity (24). Therefore, these findings indicated that CR inclusion in piglets' diets could enhance the immunomodulatory functions of piglets by modulating the pro-/anti-inflammatory cytokines and antibodies.

The gut microbiota plays a crucial role in the gastrointestinal function, immune function, and health of the host (25). Alpha-diversity is generally known as the diversity of organisms within one site or one sample. Moreover, the higher α-diversity is considered to be beneficial for the maintenance of the intestinal homeostasis of the host (26). In the present study, dietary CR or FCR supplementation had no impact on the ileal microbial community richness (Chao 1 and Simpson) and evenness (ACE and Shannon), whereas ACE, Chao 1, and OTU indexes were increased in the colon of piglets. In addition, β-diversity analysis revealed that dietary CR or FCR supplementation could significantly change the overall structure of the colonic microbial community composition of piglets. Therefore, these findings indicate that diet partially replaced with CR or FCR may contribute toward maintaining the intestinal immune homeostasis in piglets.

*Firmicutes, Bacteroidetes, Proteobacteria*, and *Actinobacteria* were the most abundant phyla in the ileum and colon of piglets at the phylum level, as these phyla are the predominant in the swine gastrointestinal tract (27, 28). Meanwhile, *Firmicutes* abundance was increased and *Bacteroidetes* was decreased in the ileum and colon of piglets after the FCR supplementation in piglet's diet. These results indicate that dietary FCR supplementation changed the structure of the intestinal microbiota in piglets. Research evidence showed that the energy absorption was enhanced in weaned piglets when the *Firmicutes* abundance was higher than that of *Bacteroidetes* (29). Similarly, the increased *Firmicutes* abundance might enhance the energy absorption of piglets fed with FCR in the present study (7). Moreover, dietary CR or FCR supplementation increased the *Actinobacteria* abundance in the ileum while decreased *Verrucomicrobia* and *Tenericutes* abundances in the ileum and colon of piglets, respectively. *Actinobacteria* is associated with the biodegradation of fiber and participate in the breakdown of plant-derived carbohydrate starch and polysaccharides, including inulin and arabinoxylan (30). Generally, a minor population of *Verrucomicrobia* was found in the intestinal microbiota in response to fermented dietary fiber intervention (7). These results were similar to those of Lu et al., (7) who reported that weaned piglets fed with the fermented corn-soybean meal had a higher *Actinobacteria* abundance and a lower *Verrucomicrobia* abundance.

At the genus level, dietary CR supplementation increased the *Escherichia-Shigella* abundance in the ileum and decreased *Terisporobacter* and *Treponema_2* abundances in the ileum and colon of piglets, respectively; whereas dietary FCR supplementation decreased *Ruminococcaceae_UCG-005* and [*Eubacterium]_coprostanoligens_group* abundances in the ileum and increased *Romboutsia* and *Rumniococcaceae_UCG-005* abundances in the colon of piglets. *Escherichia-Shigella* is known to be an opportunistic pathogen in animals and humans, which is associated with numerous disorders and infectious diseases (31). *Ruminococcaceae* plays a particularly important role in fiber degradation to obtain more energy by its digestive enzyme function (32). Further analysis of LEfSe confirmed that the impacts of dietary CR or FCR supplementation on the intestinal microbial enrichment in the ileum and colon of piglets. Taken together, dietary CR supplementation may contribute a little risk for the piglets' intestinal health, whereas the FCR supplementation could improve the intestinal health of piglets by modulating the microbiota composition in the ileum and colon.

The gut barrier functions of early-aged piglets contribute the foundation for later nutrient absorption and initial barrier protection from inflammation, which persists later in life (33). The tight junction proteins (e.g., *ZO-1, claudin-1, and occludin*), mucins, and intestinal microbiota collaboratively build up the gut barrier to maintain intestinal homeostasis and cellular functions (34). In the present study, the up-regulated mRNA expressions of tight junction proteins (*ZO-1, claudin-1*, and *occludin*) and *mucin-1* in the piglets' intestine suggested that dietary CR or FCR supplementation enhanced the intestinal physical and chemical barrier functions of piglets. In addition, the mRNA expressions of *E-cadherin, NF-*κ*B*, and *Nrf2* were up-regulated in the CR and FCR groups compared with the control group, and *NF-*κ*B* was up-regulated and *E-cadherin* was down-regulated in the FCR group compared with the CR group. Consistent with these findings, the correlation analysis also revealed that the gut barrier function parameters exhibited positive correlations with the intestinal microbial abundances of *Terisporobacter* and *Turicibacter*. Therefore, these findings suggested that dietary CR or FCR supplementation is beneficial to maintain the piglets' intestinal homeostasis.

Nutritional strategy shapes the intestinal microbiota, which impacts hepatic lipid metabolism. In addition, hepatic lipid accumulation may result from an improper balance between lipid availability and lipid disposal and thus eventually provokes lipoperoxidative stress and hepatic injury (35, 36). In the present study, dietary FCR supplementation increased the TG and ALP levels and decreased LDL-C level, which indicated that dietary FCR supplementation might cause mild inflammation in the liver, although further studies are needed to clarify this point. Interestingly, dietary CR and FCR supplementation significantly up-regulated the NF-κB gene expression in the piglets' gut in the present study. The NF-κB is a transcription factor that plays an important role in immunity and inflammation. It is also an essential factor that controls gene expression of various pro-inflammatory cytokines, chemokines, cell adhesion molecules, and acute-phase protein (37). Nevertheless, pro-inflammatory cytokines controlled by NF-κB activation (especially TNF-α) was not affected by dietary CR or FCR supplementation. However, further studies are needed for NF-κB protein evaluation in order to correlate cytokine proteins and also to clarify NF-κB activation mechanisms. The lipid accumulation in the liver also depends on the fatty acid and triglycerides synthesis and lipid metabolism-related gene expression levels. The present study found that dietary CR supplementation up-regulated the hepatic mRNA expression levels of *FASN, CEBP-*α, and *LPL* in the piglets. The *LPL* is known as the key proteolytic enzyme that takes part in lipid metabolism. The decreased level of *LPL* blocks the lipid metabolism and helps the accumulation of fat in the liver (38). Several studies showed the increasing levels of *FASN* and *CEBP-*α in the high-fat diet compared with the control group (39, 40). The reduced level of *HMG-CoA* participates in the reduction of cholesterol synthesis, which is associated with lipid metabolism, whereas *PPAR-*α and *CPT2* genes are associated with fatty acid β-oxidation (41). Moreover, the down-regulated *CYP-27A1* mRNA expression level was found to be involved with liver fibrosis (42). However, the present study showed that dietary CR or FCR supplementation up-regulated the hepatic mRNA levels of *HMG-CoA* and down-regulated *CYP-27A1* and *ACOX-1* in the piglets. Collectively, these findings indicate that dietary CR supplementation showed a potential risk and FCR supplementation had that tendency in some context. These results might be the possible reason behind the biological characteristics of CR. However, further studies are necessary to reveal the exact reason.

## Conclusion

In summary, partial replacement of corn-soybean meal with agricultural by-product CR or FCR could maintain the piglets' health *via* enhancing the intestinal and hepatic antioxidant capacity, immunomodulatory functions, and the production of IgAs. Moreover, dietary CR supplementation had more distinctive effects than the FCR on antioxidant enzymes in both intestine and liver, as well as on the production of IgA. Meanwhile, these supplements increased the gut barrier function and altered gut microbiota composition. However, dietary CR supplementation showed the potential risk to piglets' hepatic lipid metabolism, whereas microbial fermentation of CR had less impact. Nevertheless, partial replacement of corn-soybean meal with CR or FCR would be a cost-effective dietary supplemental strategy for livestock production.

## Data Availability Statement

The datasets presented in this study can be found in online repositories. The names of the repository/repositories and accession number(s) can be found in the article/Supplementary Material.

## Ethics Statement

This study was conducted following the guidelines of the Laboratory Animal Ethical Commission of the Chinese Academy of Sciences and approved by the Animal care Committee of the Institute of Subtropical Agriculture, Chinese Academy of Science, Changsha, China.

## Author Contributions

MA, HN, and XK conceived and designed the experiment. MA, HJ, YL, and PH performed the experiment. MA, HN, and YL processed the data. MA prepared and drafted the manuscript. JF and XK revised the manuscript. All authors have read and approved the final manuscript.

## Funding

This study was supported by the Special Funds for Construction of Innovative Provinces in Hunan Province (2019RS3022) and Chinese Academy of Sciences President's International Fellowship Initiative 2020PB0097.

## Conflict of Interest

The authors declare that the research was conducted in the absence of any commercial or financial relationships that could be construed as a potential conflict of interest.

## Publisher's Note

All claims expressed in this article are solely those of the authors and do not necessarily represent those of their affiliated organizations, or those of the publisher, the editors and the reviewers. Any product that may be evaluated in this article, or claim that may be made by its manufacturer, is not guaranteed or endorsed by the publisher.
