# Supplementary material for: Diets Partially Replaced With Cassava Residue Modulate Antioxidant Capacity, Lipid Metabolism, and Gut Barrier Function of Huanjiang Mini-Pigs
Source: Front Vet Sci. 2022 May 17;9:902328. doi: 10.3389/fvets.2022.902328 (PMC9152454; doi:10.3389/fvets.2022.902328)
Supplement: Supplementary file 1 [file Data_Sheet_1.docx]

**Supplementary Materials**

**Supplemental Table 1** Composition and nutrient levels of the basal diets (air-dry basis) %

| **Ingredients** | **Control group** | **CR group** | **FCR group** |
| --- | --- | --- | --- |
| Cassava residue |  | 5 |  |
| Fermented cassava residue |  |  | 5 |
| Corn gluten meal | 58.00 | 55.10 | 55.10 |
| Soybean meal | 17.65 | 16.77 | 16.77 |
| Wheat bran | 12.80 | 12.16 | 12.16 |
| Rice bran meal | 8.30 | 7.89 | 7.89 |
| Lysine | 0.20 | 0.19 | 0.19 |
| Threonine | 0.05 | 0.05 | 0.05 |
| Limestone | 0.75 | 0.71 | 0.71 |
| Dicalcium phosphate | 0.95 | 0.90 | 0.90 |
| NaCl | 0.30 | 0.29 | 0.29 |
| Premix ^a^ | 1.00 | 0.95 | 0.95 |
| **Total** | **100** | **100** | **100** |
| Nutrient level **^b^** |  |  |  |
| Dry matter | 95.13 | 95.26 | 94.84 |
| Crude fiber | 7.57 | 6.75 | 6.24 |
| Digestible energy (MJ/kg) | 16.99 | 17.13 | 17.07 |
| Non-digestible fiber | 22.13 | 24.38 | 31.22 |
| Acid detergent fiber | 8.14 | 7.98 | 10.83 |
| Crude protein | 9.76 | 8.81 | 14.76 |
| Crude ash | 6.29 | 5.80 | 5.62 |

^a^ The premix provided per kilogram of control group diet: copper sulphate 20 mg, ferrous sulfate 49.50 mg, zinc sulfate 29.70 mg, manganese sulphate 19.80 mg, iodine 39.60 μg, selenium 39.60 μg, cobalt 19.80 μg, VA 16087.39 IU, VD_3_ 4949.97 IU, VE 39.60 IU, VK_3_ 4.95 mg, VB_1_ 4.95 mg,VB_2_ 12.37 mg,VB_6_ 3.96 mg,VB_12_ 0.037 mg, D-biotin 0.30 mg, D-pantothenic acid 24.75 mg, Folic acid 2.47 mg, niacinamide 49.50 mg.

^b^ Data are the results of chemical analysis conducted in triplicate

**Supplemental Table 2** Primers sequences used for real-time polymerase chain reaction

| **Gene** | **5′ - Primer (F)** | **3′ - Primer (R)** | **Accession No.** | **Size (bp)** |
| --- | --- | --- | --- | --- |
| β-actin | GATCTGGCACCACACCTTCTACAAC | TCATCTTCTCACGGTTGGCTTTGG | XM_021086047.1 | 107 |
| *ZO-1* | CCTTCCCAGTCTCCTTGCTG | GAGGGCTGGTCTCAAACTCC | XM_021098783.1 | 187 |
| *Claudin-1* | AGCTATGGCCAACGCGG | TGCTTGCAAAGTGGTGTTCAG | NM_001244539.1 | 241 |
| *Occludin* | CAGGTGCACCCTCCAGATTG | ATGTCGTTGCTGGGTGCATA | NM_001163647.2 | 167 |
| *Mucin-1* | GTGCTTACAGGTGAGGGGC | ACAGATCCTGGCCTGAACTT | XM_021089730.1 | 143 |
| *E-cadherin* | CGACGGTGTGGTTACAGTCA | AGAGGGAGAGTCCTGATGGC | NM_00163060.1 | 163 |
| *Keap1* | CCCATGAGCGTACCACGAAA | CTCGGGTTCATACCTCTCCAC | XM_005654811.3 | 117 |
| *NF-κB* | GCTGGAATGAAGCACGGAAC | GCAAGTTGCATGGCCTTCTC | NM_001048232.1 | 236 |
| *TLR4* | GACAGCAATAGCTTCTCCAGC | GGTTTGTCTCAACGGCAACC | NM_001113039.2 | 205 |
| *Nrf2* | TCCATTCCAGAATTACAGTGTCT | TGCTGCTGAAGGAATCCTCG | XM_013984303.2 | 193 |
| *CD36* | GTCAACCTATTGGTCAAACCAG | TCATCACCAATTGTACCAGTCTCA | NM_001044622.1 | 110 |
| *FASN* | TCCAAGGAGCAAGGTGTGAC | CCCATGTTCGACTTGGTGGA | NM_001099930.1 | 224 |
| *CEBP-α* | GGCAAAGCCAAGAAGTCGGTA | TCCAGCACCTTCTGTTGAGTC | XM_003127015.4 | 134 |
| *LPL* | GGCCACGTTTATCGACTGGA | CAGCTGGATCTAGGCCAGTA | NM_214286.1 | 152 |
| *DGTA1* | TTTCAGCAACTACCGTGGCA | ACCAGGATGCCGTACTTGAT | NM_214051.1 | 102 |
| *SREBP-1c* | CCCTCGTCTACCACAAGCTG | CTCAGGAAGAAGCGGGTCAG | NM_214157.1 | 220 |
| *HMG-CoA* | ATTGCCTGTGGTCAGGATGC | CCAGGATTGTCTTTGCACGC | NM_001122988.1 | 212 |
| *CYP-27A1* | CCAAGAGGGCAAGTACCCAG | CCATGACTCTCCTTCCGTGG | NM_001243304.1 | 106 |
| *CPT2* | GATGGCCAGTTCAGGAAAACAG | AATGGGGTTTCGAGCAGTCA | NM_001246243.1 | 162 |
| *PPAR-α* | AGCAATAACCCGCCTTTCGT | GTAGACGCCGTACTTCAGCA | NM_001044526.1 | 243 |
| *ACOX-1* | ACGTGGTCGTCAAGCTCTTT | CTGTCATGATGCTCCCCTGA | NM_001101028.1 | 147 |

*ZO-1*, zonula occludens-1; *Keap1*, kelch-like ECH-associated protein 1; *NF-κB*, nuclear factor kappa B; *TLR4*, toll like receptor 4; *Nrf2*, nuclear factor erythroid 2-related factor 2; *FASN,* fatty acid synthase; *CEBP-1α*, CCAAT enhancer-binding protein alpha; *LPL*, lipoprotein lipase; *DGTA1*, diacylglycerol-o-acyltransferase 1; *SREBP-1c*, sterol regulatory element-binding protein 1c; *HMG-CoA*, 3-hydroxy-3-methylglutaryl-CoA reductase; *CYP-27A1*, cytochrome p450 family 27 sub-family A member 1; *CPT2*, carnitine palmitoyl transferase 2; *PPAR-α*, peroxisome proliferator activated receptor alpha; *ACOX-1*, acetyl-CoA oxidase 1.
